# Recent Advances of the NLRP3 Inflammasome in Central Nervous System Disorders

**DOI:** 10.1155/2016/9238290

**Published:** 2016-08-29

**Authors:** Keren Zhou, Ligen Shi, Yan Wang, Sheng Chen, Jianmin Zhang

**Affiliations:** ^1^Department of Neurosurgery, Second Affiliated Hospital, School of Medicine, Zhejiang University, Hangzhou, Zhejiang, China; ^2^Brain Research Institute, Zhejiang University, Hangzhou, Zhejiang, China

## Abstract

Inflammasomes are multiprotein complexes that trigger the activation of caspases-1 and subsequently the maturation of proinflammatory cytokines interleukin-1*β* and interleukin-18. These cytokines play a critical role in mediating inflammation and innate immunity response. Among various inflammasome complexes, the NLRP3 inflammasome is the best characterized, which has been demonstrated as a crucial role in various diseases. Here, we review recently described mechanisms that are involved in the activation and regulation of NLRP3 inflammasome. In addition, we summarize the recent researches on the role of NLRP3 inflammasome in central nervous system (CNS) diseases, including traumatic brain injury, ischemic stroke and hemorrhagic stroke, brain tumor, neurodegenerative diseases, and other CNS diseases. In conclusion, the NLRP3 inflammasome may be a promising therapeutic target for these CNS diseases.

## 1. Introduction

In the central nervous system (CNS), the innate immune response plays a significant role in the pathology after tissue damage or pathogen invasion. This process is known as neuroinflammation and is characterized by the activation of the microglia and astrocyte population [1]. It is known that several cell types in the brain express specialized pattern recognition receptors (PRRs) such as membrane-bound Toll-like receptors (TLRs) and cytosolic NOD-like receptors (NLRs). The NOD-like receptors are a class of cytosolic sensors or receptors that respond to a variety of pathogen-associated molecular patterns (PAMPs) linked to various microbes and damage-associated molecular patterns (DAMPs) produced during tissue-based injury [2].

One of the most extensively studied classes of NLRs is the inflammasome-forming NLRs including NLRP1, NLRP3, NLRC4, NLRC5, NLRP6, NLRP7, and NLRP12 as well as the non-NLR inflammasome receptor known as AIM2. Among them, NLRP3 is the best characterized. The NLRP3 inflammasome is composed of NLRP3, apoptosis-associated speck-like (ASC) adapter protein, and the downstream effector enzyme (procaspase-1) [3]. When stimulated by PAMP or DAMP, NLR forms a protein complex known as the inflammasome through the combination of the adaptor protein ASC [4]. This initiates the cleavage of procaspase-1 into the active and mature form of caspase-1 [5]. Subsequently, active caspase-1 converts the inactive pro-IL-1*β* and pro-IL-18 into their active and secreted forms: IL-1*β* and IL-18. These cytokines initiate or amplify diverse downstream signaling pathways and drive proinflammatory responses, leading to cellular damage, such as autophagy and pyroptosis [6, 7].

Recently, increasing attention is being paid to the role of the NLRP3 inflammasome in the central nervous system (CNS). The NLRP3 inflammasome plays a pathogenic role in neuroinflammatory diseases. Here, we review described mechanisms that have been proposed to be involved in the activation and regulation of NLRP3 inflammasome and further explore the role of NLRP3 inflammasome in several CNS diseases.

## 2. The Activation and Regulation of NLRP3 Inflammasome (Figure 1)

To date, it has been demonstrated that the activation of the NLRP3 inflammasome appears to occur by two signals. The initial priming signal, induced by the Toll-like receptor (TLR)/nuclear factor NF-*κ*B pathway, affects NLRP3 at the transcriptional level and also serves to trigger posttranslational modifications of inflammasome components [8, 9]. The second signal triggers assembly of the NLRP3 inflammasome complex [10, 11]. The NLRP3 inflammasome complex can be activated by both exogenous (including infection, tissue damage, and metabolic dysregulation) [12] and endogenous molecules such as extracellular ATP, hyaluronan, A*β* fibrils, and uric acid crystals [13].

For a large number and diversity of NLRP3 inflammasome stimuli, it seems unlikely that they all bind to the NLRP3 structure to form the NLRP3 inflammasome. So far, there have been three mainly mechanisms regarding activation of the NLRP3 inflammasome, including the generation of reactive oxygen species (ROS), the efflux of potassium, and the rupture of lysosomal [14, 15].

### 2.1. Reactive Oxygen Species (ROS)

ROS, mainly associated with the normal or malfunctioning mitochondria, has been proved to play a significant role in the activation of NLRP3 inflammasome [16]. Numerous NLRP3 inflammasome activators are known to trigger mitochondrial ROS production.

A recent study has shown that the thioredoxin-interacting protein (TXNIP) is a ROS-sensitive regulator of the activation of NLRP3 inflammasome [17]. The binding of TXNIP to NLRP3 leads to the activation of NLRP3, the recruitment of ASC and procaspase-1, and the formation of the active NLRP3 inflammasome complex [18, 19]. In addition, several researches suggest that the damage to NADPH oxidase by mitochondrial ROS can activate the inflammasome [20, 21]. Other studies suggest that NADPH oxidase and the production of ROS are dispensable for NLRP3 inflammasome activation, but crucial for IL-1*β* secretion [15, 22]. Moreover, it has been demonstrated that the mitochondria-targeted antioxidant Mito-TEMPO, which targets mitochondrial ROS, can inhibit the activation of inflammasome and subsequently reduce the secretion of IL-1*β* and IL-18 [23, 24].

However, ROS activation is not an absolute requirement for activation of all NLRP3 inflammasomes [25]. Some studies implicate that mitochondrial ROS mediates the upregulation of NLRP3 and pro-IL-1*β* transcription rather than the NLRP3 inflammasome activation [26]. Therefore, more studies are required to understand the precise role of ROS in regulating NLRP3 inflammasome activation.

### 2.2. K^+^ Efflux

Another fully studied mechanism of NLRP3 inflammasome activation is the decrease in the intracellular K^+^. Several mechanisms underlying the efflux of K^+^ have been proposed. For example, high extracellular ATP concentrations can reduce intracellular K^+^ concentrations by activating the P2X purinergic receptor 7 (P2X7), which is considered an important signaling pathway to activate NLRP3 inflammasomes [27, 28]. Furthermore, a reduction in intracellular K^+^ levels was found to be essential for NLRP3 inflammasome activation when triggered by bacterial infection, MSU crystals, and pore-forming toxins [29, 30]. Another study indicated that inflammasome activation in response to many NLRP3 activators was effectively inhibited by K^+^ channel inhibitor glibenclamide [13]. Recently, the reduction in intracellular K^+^ concentration is thought to be a common pathway for NLRP3 inflammasome complex activation. However, the mechanisms of how low cytoplasmic K^+^ concentration activates inflammasome activation are needed to further study.

### 2.3. Rupture of Lysosome

It has also been widely accepted that the disruption of the lysosomal membrane can result in NLRP3 activation. lysosome destabilization, caused by the phagocytosis of specific crystalline and particulate matter, leads to the release of lysosomal contents [31]. Cathepsin B, as one of the lysosomal contents, has been proved to activate the NLRP3 inflammasome. Cathepsin B inhibitor CA-074-Me was found to partially inhibit NLRP3 activation [32, 33]. Besides the mechanisms of activation by endogenous crystalline, the role of environmentally derived crystals such as asbestos, silica, and aluminum salts has also been proved in NLRP3 inflammasome activation [34]. In addition, to understand how lysosomal rupture leads to NLRP3 activation, a recent study found that the TAK1-JNK pathway, a MAPK signaling pathway, was activated through lysosome rupture and that this activation was necessary for the complete activation of the NLRP3 inflammasome [35]. Thus, the lysosome plays an essential role in the activation of the NLRP3 inflammasome. Further understanding of the mechanism is needed in future.

### 2.4. Other Activators

Despite the former three mechanisms regarding activation of the NLRP3, it is becoming increasingly clear that one signal alone is insufficient to induce inflammasome activation [16]. Several other pathways have been revealed to explain the mechanisms by which diverse stimuli activate the NLRP3 inflammasome complex. Recently, it has been reported that Ca2+ mobilization mediated mitochondrial damage and dysfunction can also activate the NLRP3 inflammasome [36, 37]. In addition, mitochondria-associated cardiolipin is required for recruitment and activation of the NLRP3 inflammasome [38]. Moreover, some studies elucidated the contribution of the mitochondrial antiviral signaling protein (MAVS) in NLRP3 inflammasome activation [39, 40]. It has also been reported that infection with RNA virus initiates assembly of the RIP1–RIP3 complex, which promotes activation of the GTPase DRP1 and its translocation to mitochondria to drive mitochondrial damage and activation of the NLRP3 inflammasome [41].

### 2.5. Negative Regulation

Despite the multiple positive regulation contributed to the activation of NLRP3 inflammasome, negative regulation is also necessary to maintain appropriate induction of inflammasome function.

Recent studies have indicated that autophagy can act as a negative regulator of NLRP3 inflammasome activation by removing sources of endogenous NLRP3 inflammasome agonists [42, 43], suppressing of IL-1*β* secretion [44], and degrading inflammasome components, such as NLRP3 and ASC [45, 46]. And also inhibition of autophagy by 3-methyladenine (3-MA) can promote the activation of the NLRP3 inflammasome [47]. Additionally, nitric oxide (NO) downregulates NLRP3 activation through enhancing the removal of the dysfunctional mitochondria and preventing assembly of the inflammasome [48, 49]. Moreover, type I IFNs inhibit the NLRP3 inflammasome in both the priming signal and the activation signal [50]. Other forms of negative regulation, such as the roles of microRNAs and bacterial and viral mechanisms, have been detailed in recent reviews [8, 51].

Considering the recent findings of activation and regulation of the NLRP3 inflammasome, further understanding of these molecular mechanisms and signal pathways may be helpful in designing potential therapeutics to prevent inflammatory diseases associated with the NLRP3 inflammasome.

## 3. NLRP3 Inflammasome and CNS Diseases

Recently, an increasing number of studies have been investigating the underlying role of NLRP3 inflammasome in the central nervous system. The NLRP3 inflammasome has been proved to express in diversity of cells such as microglia, astrocyte, neuron, and endothelial cell [52–55] and in different kinds of diseases such as traumatic brain injury, stroke, brain tumor, neurodegenerative disease, and others.

### 3.1. NLRP3 Inflammasome and Traumatic Brain Injury

Traumatic brain injury (TBI), caused by physical force to the brain tissue, initiates a primary insult and secondary cascade of events. The primary insult results in direct neuronal loss and necrotic death, which is then followed by a wave of injury cascades including excitotoxicity, oxidative stress, mitochondrial dysfunction, blood-brain barrier disruption, and inflammation [56]. Numerous studies laid emphasis on the role of inflammatory response among the components of the secondary brain injury [57, 58].

The significant increasing of IL-1*β* and IL-18 after experimental TBI has been demonstrated [59]. Recently, a study found that TBI could induce assembly of NLRP3 inflammasome complex, expression of ASC, activation of caspase-1, and processing of IL-1*β* and IL-18. It may be possible that NLRP3 inflammasome is a promising therapeutic target for patients with TBI [60]. Similar to NLRP3, NLRP1 has also been proved as an important component of the innate inflammatory response after TBI. TBI induced assembly of the NLRP1 inflammasome, cleavage of X-linked inhibitor of apoptosis protein (XIAP), activation of caspase-1, and processing of IL-1*β* [61]. Another study showed that TBI patients with higher levels of NLRP1 in the cerebrospinal fluid (CSF) had a worse outcome than that with a lower expression of NLRP1 [62]. NLRP1 and NLRP3 may work together to play a role in the inflammation after TBI and serve as candidate therapeutic targets in TBI [4]. However, the relationship between those two inflammasomes needs further study.

Although the studies indicated a correlation between enhanced inflammasome expression and TBI pathology, the potential functional role that inflammasome may play in TBI remains to be directly demonstrated. Therefore, inflammatory response, especially in the postinjury period of TBI, is an important therapeutic target for reducing the neurological dysfunction and improving the outcome.

### 3.2. NLRP3 Inflammasome and Ischemia Stroke

Stroke is regarded as a severe disorder with high mortality and long-term disability. Clinically, stroke can be classified into ischemic stroke and hemorrhagic stroke. Ischemic stroke commonly accounts for approximately 80% of all stroke cases while hemorrhagic stroke accounts for approximately 20% [63]. The mechanisms responsible for ischemic stroke-induced neuronal cell death include bioenergetic failure, oxidative stress, excitotoxicity, apoptosis, and inflammatory process [64–66].

Recently, the increasing number of evidences has indicated that inflammatory mechanism fundamental to the innate immune system may contribute to the death of neuronal and glial cell during cerebral ischemia [67]. The NLRP3 inflammasome has been proved to play an important role in detecting cellular damage and mediating inflammatory responses to tissue injury during ischemic stroke. Hence, targeting pathways upstream and downstream of NLRP3 inflammasome signaling may offer substantial promise in developing new therapeutics for stroke [68].

A study found the neuroprotective effects of intravenous immunoglobulin (IVIg), which could significantly reduce the levels of NLRP3 inflammasome proteins as well as IL-1*β* and IL-18 during simulated ischemia in vitro and in a mouse model of focal ischemic stroke [69]. Another study showed that intermittent fasting (IF) could contribute to reducing expression of NLRP1 and NLRP3 inflammasome and the precursors of IL-1*β* and IL-18 in a mouse model of focal ischemic stroke by suppressing the activation of NF-*κ*B and MAPK pathway [70]. More evidence showed that, in neuronal cells, oxygen-glucose deprivation (OGD) could induce the accumulation of dsRNA to prime the NLRP3 and proinflammatory cytokines production, which were involved in the inflammatory progression and injuries of cerebral ischemia [71]. These suggest that targeting NF-*κ*B pathway and transcriptional level may provide a therapeutic effect on inflammasome expression and activity during cerebral ischemia. Recently, the research targeting TXNIP/NLRP3 inflammasome activation is also a hotspot. A study indicated that Umbelliferone (UMB) suppressed the inflammatory cytokines production through the inhibition of TXNIP/NLRP3 inflammasome activation [72]. Another study showed that curcumin inhibited TXNIP/NLRP3 inflammasome activation by suppressing endoplasmic reticulum stress and thereby protected neuronal cell survival from glutamate neurotoxicity after ischemic insult [73]. In addition, one study demonstrated that pharmacological inhibition by using resveratrol or genetic deletion of TXNIP attenuated brain infarction and neurological outcome in mice embolic mode via restoring redox-balance and inhibition of TXNIP-NLRP3 inflammasome activation [74]. Moreover, there are also researches targeting inflammasome components NLRPs, ASC, and caspase-1. One study demonstrated that estrogen and progesterone could regulate ASC and NLRP3 at the protein level and reduce the expression of inflammasome components in the transient focal rat ischemic model [53]. In addition, Bruton's tyrosine kinase (BTK) regulated activation of the NLRP3 inflammasome by interacting with NLRP3 and ASC [75]. Another study found that A151, a synthetic oligodeoxynucleotide, attenuated ischemic brain injury by reducing the maturation of caspase-1 and IL-1*β* and the production of NLRP3 [76].

Therefore, numerous studies have focused on the expression, activity, and products of NLRP3 inflammasome, which may discover potential therapeutics for ischemia stroke. However, there are still many underlying mechanisms of the inflammasome remained to investigate.

### 3.3. NLRP3 Inflammasome and Hemorrhagic Stroke

Spontaneous intracerebral hemorrhage (ICH), as one type of hemorrhagic stroke, is also a devastating disease with high morbidity and mortality [77]. The mechanisms responsible for neurological dysfunction after ICH include hematoma formation, brain edema, inflammation, and microglia activation [78–81]. Accumulating evidence indicates that inflammatory mechanism, especially the role of NLRP3 inflammasome, is involved in the pathophysiology of ICH [82].

In a mouse model of ICH, Ma et al. found the role of NLRP3 inflammasome in contributing to ICH-induced inflammatory activation. The mPTP inhibitor (TRO-19622) and mitochondria ROS scavenger (Mito-TEMPO) were used in the study to indicate the mechanism of mitochondria ROS in NLRP3 inflammasome activation [24]. The inhibition of P2X7R pathway by using blue brilliant G (BBG) could be a potential therapeutic target for secondary brain injury after ICH [83]. Additionally, a recent study found that a recombinant adenovirus encoding NLRP3 RNAi attenuated inflammation in ICH [84]. Moreover, another study identified miR-223 suppressed NLRP3 production by directly binding to its 3′UTR, which reduced neuronal inflammation and improved neuronal function after ICH [85]. Recent studies have revealed new information on the NLRP3 inflammasome during ICH, and the NLRP3 inflammasome may be a promising therapeutic target in ICH patients.

Subarachnoid hemorrhage (SAH), as another type of hemorrhagic stroke, is associated with a high mortality and morbidity [86, 87]. Recently, increasing evidence has emphasized the role of early brain injury (EBI) associated with the poor outcome of SAH patients [88–91]. The underlying mechanisms include a reduction in cerebral blood flow, increased intracranial pressure, oxidative stress, apoptosis, and inflammation [92]. NLRP3 inflammasome has been proved a key component of inflammatory response in the pathophysiology of SAH.

A recent study made the observation that treatment with minocycline in a rat SAH model could inhibit NLRP3 inflammasome activation and attenuate brain edema in EBI after SAH. This effect may be associated with the reduction of mitochondrial ROS [93]. Another study demonstrated the downregulating role of hydrogen-rich saline (HS) treatment in the activation of NLRP3 inflammasome via inhibiting NF-*κ*B pathway after SAH [94]. In addition, the neuroprotection role of melatonin treatment has been proved in the EBI following SAH by inhibiting NLRP3 inflammasome activation and NLRP3-associated apoptosis [95].

An increasing number of studies focus on the role of NLRP3 inflammasome in inflammatory response after SAH. These studies may provide a promising therapeutic choice for patients with SAH.

### 3.4. NLRP3 Inflammasome and Brain Tumor

In recent years, the important role of NLRP3 inflammasome in carcinogenesis and tumor progression has been reported. A number of endogenous and exogenous stimuli can behave as tumor promoters by inducing chronic inflammation and consequently provide signals for inflammasome activation in cancer [96]. And NLRP3 inflammasome is also reported to suppress the function of natural killer cell in controlling carcinogenesis and metastases [97]. Moreover, NLRP3 inflammasome plays a significant role in tumor control by recruiting neutrophils, which may provide a prognostic marker and promising therapeutic target in cancer patients [98].

In CNS, malignant glioma is the most common primary brain tumors with poor prognosis. Increasing evidence indicates the crucial role of innate immunity and chronic inflammation in carcinogenesis and tumor progression [99–101]. And the role of NLRP3 inflammasome in glioma has also been described. NLRP3 inflammasome can active in glioblastoma multiforme (GBM) cells constitutively [102]. NLRP3 inflammasome can also be activated by different signals in different types of cells [54, 55, 103]. Moreover, by using a mouse glioblastoma model, it has been proved that the inhibition of NLRP3 can reduce tumor growth and prolong the survival of mouse following IR treatment. They demonstrated that NLRP3 inflammasome was a molecular link between brain aging and progression of glioma and radiotherapy resistance [104].

The NLRP3 gene signature may serve as a promising biomarker in glioma patients. However, the potential mechanism of NLRP3 inflammasome in the progress of brain tumor has not been totally understood. More studies are needed in understanding the character of inflammasome and exploring therapeutic potential in brain cancer.

### 3.5. NLRP3 Inflammasome and Neurodegenerative Diseases

Recent researches have indicated that innate immune activation and neuroinflammation may be involved in various neurodegenerative diseases, such as Alzheimer's disease (AD) and Parkinson's disease (PD). There is increasing evidence that sustained inflammatory responses may contribute to disease progression. It can not only be a consequence but also be a trigger of pathology [105]. Neuroinflammatory cascades rely on the activation of NLRP3 inflammasome, which has been proved crucial in neurodegenerative diseases.

AD is the most prevalent form of dementia. Extensive evidence has indicated that IL-1*β* and IL-18 may contribute to the pathogenesis of AD and cause cognitive impairment [106, 107]. The pathogenesis of AD involves extracellular accumulation of amyloid-*β* (A*β*) in senile plaques [108]. It has been demonstrated that the toxicity of A*β* can activate NLRP3 inflammasome, process IL-1*β* and IL-18, and eventually induce AD pathology and tissue damage. Moreover, in AD transgenic mouse model, the inhibition of NLRP3 can largely protect memory loss and decrease A*β* deposition, which provides a possibility of AD therapy by targeting NLRP3 inflammasome [109].

PD is another common degenerative disease with two main pathological hallmarks: progressive loss of dopaminergic neurons in substantia nigra and accumulation of Lewy body in neurons [110]. Lewy body is mainly composed of the presynaptic protein *α*-synuclein (*α*-syn), which has been proved to be a significant role in NLRP3 inflammasome activation and PD pathogenesis [111, 112]. And it also provides a therapeutic possibility for PD.

In addition, numerous researches have revealed the role of NLRP3 inflammasome in many other neurodegenerative diseases such as frontotemporal dementia, amyotrophic lateral sclerosis (ALS), Huntington's disease, and multiple sclerosis (MS) [105, 113]. The innate immune activation could be an early cause in neurodegenerative diseases and this indicates that anti-inflammatory therapies could be a promising treatment approach. Although the inhibition of inflammation may not alter the underlying cause of disease, it may reduce the production of factors involved in neurotoxicity and consequently result in clinical benefits.

### 3.6. NLRP3 Inflammasome and Other CNS Diseases

It has been widely accepted that neuroinflammation is involved in the epileptogenesis by promoting neuronal excitability and decreasing seizure threshold [114]. The role of the NLRP3 inflammasome in status epilepticus (SE), one of the most serious types of epilepsy, has been proved. In SE rat model, the expression of NLRP3 inflammasome was promoted, which could activate caspase-1 signaling and contribute to neuroinflammation and epileptogenesis. The inhibition of NLRP3 inflammasome may play a neuroprotective role against neuroinflammation and neuronal damage followed by SE [115].

In addition, increasing evidence shows that the NLRP3 inflammasome is also involved in microbial infections in CNS. Microglial cells constitute the first defense line of the CNS against microbial invasion [116]. A study of Jamilloux et al. found that microglial cells detected that legionella pneumophila could lead to the activation of inflammasome [117]. And another study showed that NLRP3 knockout mice infected with pneumococcal meningitis could present decreased scores of disease severity and brain inflammation [118]. Moreover, another study found the expression of NLRP3 and IL-1*β* changed in the brain of avian influenza virus H9N2 infected mice. This study indicated the role of NLRP3 inflammasome in host response to influenza virus infection and the outcome of pathological injury and clinical manifestation [119]. In general, inhibiting inflammasome activation might be a promising target for microbial infection diseases. Besides, many other CNS disorders, such as prion diseases, experimental autoimmune encephalomyelitis (EAE), are associated with the activation of NLRP3 inflammasome [120, 121]. Further studies are aimed at providing new therapeutic choices for all these disorders.

## 4. Conclusion

In this review, we have elaborated on the mechanisms involved in the activation and regulation of NLRP3 inflammasome. In addition, we collected the recent researches on the role of NLRP3 inflammasome in CNS diseases. The exact molecular mechanisms on the assembly, activation, and regulation of NLRP3 inflammasome are required to be further examined, which are very important for NLRP3 inflammasome to be as a novel therapeutic strategy in CNS disorders.

## Figures and Tables

**Figure 1:**
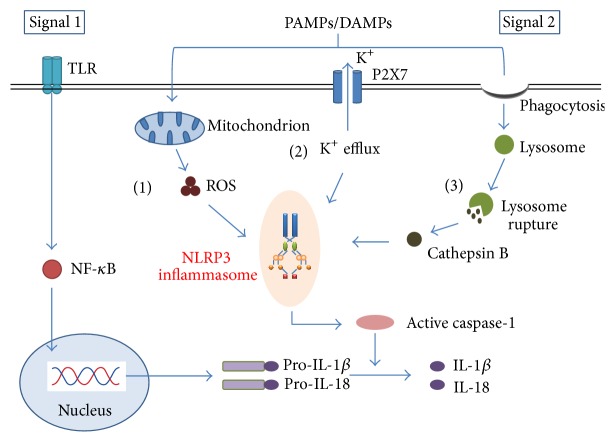
Models of NLRP3 inflammasome activation. Signal 1 activates the Toll-like receptor (TLR)/NF-*κ*B pathway, leading to the transcription of pro-IL-1*β* and pro-IL-18. Signal 2 is mediated by PAMPs or DAMPs stimulation and promotes the assembly of NLRP3 inflammasome complex. Three main mechanisms of the NLRP3 inflammasome activation have been proposed. (1) Stimuli can trigger the production of mitochondrial ROS and then induce the NLRP3 inflammasome activity. (2) Extracellular ATP or bacterial toxins can induce K^+^ efflux through the P2X purinergic receptor 7 (P2X7), which leads to the activation of the NLRP3 inflammasome. (3) The phagocytosis of specific crystalline can cause lysosomal rupture and induce the release of lysosomal contents cathepsin B, which contributes to the activation and assembly of the NLRP3 inflammasome. Consequently, these trigger the cleavage of procaspase-1 into its active and mature form caspase-1, which leads to the production of the mature IL-1*β* and IL-18.
